# The potential association between metabolic disorders and pulmonary tuberculosis: a Mendelian randomization study

**DOI:** 10.1186/s40001-024-01845-0

**Published:** 2024-05-09

**Authors:** Zhi-xiang Du, Yun-yao Ren, Jia-luo Wang, Shun-xin Li, Yi-fan Hu, Li Wang, Miao-yang Chen, Yang Li, Chun-mei Hu, Yong-feng Yang

**Affiliations:** 1 Department of Infectious Disease and Liver Disease, The Second Hospital of Nanjing, Nanjing University of Chinese Medicine, Nanjing, Jiangsu Province 210003 China; 2 Department of Tuberculosis, The Second Hospital of Nanjing, Nanjing University of Chinese Medicine, Nanjing, Jiangsu Province 210003 China; 3 Department of Infectious Diseases, The Affiliated Taizhou People’s Hospital of Nanjing Medical University, Taizhou School of Clinical Medicine, Nanjing Medical University, Taizhou, Jiangsu Province China

**Keywords:** Mendelian randomization, Pulmonary tuberculosis, Metabolic disorders, Hyperglycemia, Dyslipidemia

## Abstract

**Background:**

Metabolic disorders (MetDs) have been demonstrated to be closely linked to numerous diseases. However, the precise association between MetDs and pulmonary tuberculosis (PTB) remains poorly understood.

**Method:**

Summary statistics for exposure and outcomes from genome-wide association studies (GWASs) for exposures and outcomes were obtained from the BioBank Japan Project (BBJ) Gene–exposure dataset. The 14 clinical factors were categorized into three groups: metabolic laboratory markers, blood pressure, and the MetS diagnostic factors. The causal relationship between metabolic factors and PTB were analyzed using two-sample Mendelian Randomization (MR). Additionally, the direct effects on the risk of PTB were investigated through multivariable MR. The primary method employed was the inverse variance-weighted (IVW) model. The sensitivity of this MR analysis was evaluated using MR-Egger regression and the MR-PRESSO global test.

**Results:**

According to the two-sample MR, HDL-C, HbA1c, TP, and DM were positively correlated with the incidence of active TB. According to the multivariable MR, HDL-C (IVW: OR 2.798, 95% CI 1.484–5.274, *P* = 0.001), LDL (IVW: OR 4.027, 95% CI 1.140–14.219, *P* = 0.03) and TG (IVW: OR 2.548, 95% CI 1.269–5.115, *P* = 0.009) were positively correlated with the occurrence of PTB. TC (OR 0.131, 95% CI 0.028–0.607, *P* = 0.009) was negatively correlated with the occurrence of PTB. We selected BMI, DM, HDL-C, SBP, and TG as the diagnostic factors for metabolic syndrome. DM (IVW, OR 1.219, 95% CI 1.040–1.429 *P* = 0.014) and HDL-C (IVW, OR 1.380, 95% CI 1.035–1.841, *P* = 0.028) were directly correlated with the occurrence of PTB.

**Conclusions:**

This MR study demonstrated that metabolic disorders, mainly hyperglycemia, and dyslipidemia, are associated with the incidence of active pulmonary tuberculosis.

**Supplementary Information:**

The online version contains supplementary material available at 10.1186/s40001-024-01845-0.

## Introduction

Before the emergence of coronavirus disease (COVID-19), tuberculosis (TB) was the most lethal infectious disease globally caused by a single infectious agent. According to the Global Tuberculosis Report of 2023, more than 10 million people die from TB annually [1]. Epidemiologic studies indicate that a quarter of the global population is infected with Mycobacterium TB, but only 5% of the infected people will eventually develop TB in the first 2 years [2]. The activation of TB is a complex process affected by multiple risk factors, such as smoking, diabetes mellitus (DM), alcohol use, undernutrition, and social determinants [3]. In 2022, TB patients in China accounted for 7.1% of global TB cases, second only to those in India and Indonesia among the 30 countries with a high-burden [1]. The complexity of tuberculosis onset affects the prevention of the tuberculosis epidemic and the outcome of antituberculosis treatment. As a result, China has made remarkable progress in reducing the number of TB cases over the past three decades, but there is still a long way to go to meet the World Health Organization's strategy to end TB by 2030[4].

Metabolic diseases are a growing global health challenge, representing a group of pathological conditions clustered by several diseases such as type 2 diabetes, hypertension, hyperlipidemia, obesity and nonalcoholic fatty liver disease (NAFLD) [5]. Individuals with diabetes or insulin resistance combined with more than two factors of metabolic disorders (MetDs), including obesity, dyslipidemia, hypertension, or microalbuminuria, were defined as having metabolic syndrome (MtsD) by the WHO at the end of the last century [6]. In 2001, the Adult Treatment Panel III of the US National Cholesterol Education Program (NCEP-ATP III), which consists of five components, proposed diagnostic criteria for the clinical diagnosis of high-risk individuals. In 2006, the International Diabetes Federation (IDF) set the waist circumference to > 94 cm for men or > 80 cm for women. There is a certain difference in the three most popular definitions of MtsD, such as the definition of blood glucose by the WHO, which is more than 6.1 mmol/L (110 mg/dl), and the NCEP's recommendation, which is greater than 5.6 mmol/L (110 mg/dl) [7]. The baseline level of body mass index (BMI) of Chinese individuals is lower than that of individuals of other races (baseline value, 21; mean, 18.5 to 23.9) [8]. Therefore, the Joint Committee for Developing Chinese Guidelines (JCDCG) proposed the definition of MetS for the Chinese population in 2016 [9]. A national cross-sectional study from 2012 to 2015 reported the prevalence of MetS in the Chinese population based on the definitions of IDF, the revised ATP III, and the JCCDS. The diagnosis results were consistent in men, but the prevalence results were quite different in women. MtsD has been confirmed to be a common disease in China. The JCCDS diagnostic criteria for MtsD are more suitable for the Chinese population [10].

With the increasing attention given to diseases related to MetDs, the relationship between these diseases and the occurrence of tuberculosis is gradually emerging. T2DM is confirmed to have a well-documented association with active tuberculosis. More critically, DM also increases the risk of latent tuberculosis infection (LTBI) [11]. A systematic review indicated that no evidence supports an association between TB and hypertension. Nonetheless, the authors confirm that the need for properly designed studies will likely affect the results' authenticity of the results [12]. Patients with TB are generally malnourished. However, a study from China indicated that overweight individuals are at risk of developing tuberculosis [13]. Hsien-Ho Lin's study indicated that the relationships between obesity, diabetes, and the risk of tuberculosis were complex and nonlinear [14]. The relationship between abnormal cholesterol metabolism and the risk of active TB is controversial. In a Korean study, 32,078 cases of tuberculosis occurred among 5,000,566 subjects after 8.2 years of follow-up COX risk regression analyses confirmed a significant negative correlation between total cholesterol levels and the risk of tuberculosis. A cohort study in Singapore confirmed that a high cholesterol diet was positively associated with tuberculosis risk. Whether low total cholesterol contributes to TB or is a consequence of the disease is unclear [15]. Therefore, more studies are needed to explore the causal relationship between MetDs and TB.

Mendelian randomization (MR) is applied to assess the causality between a modifiable exposure and a clinically relevant outcome. Genome-wide association studies (GWASs) are nonmodifiable and are used as instrumental variables to examine exposure–outcome causal relationships [16]. Undoubtedly, randomized, controlled trials (RCTs) are the gold standard for establishing a causal relationship. When clinical research costs are too high, impractical, or immoral, MR research has become an excellent alternative to RCTs [17]. Considering the lack of studies exploring the connection between MetDs and TB, we performed two-sample univariate and multivariable MR investigations in this research to assess the potential causal relationship between MetDs and the risk of PTB, thereby providing a theoretical basis for the prevention and management of tuberculosis.

## Materials and methods

### Study design and data sources

**Fig. 1:**
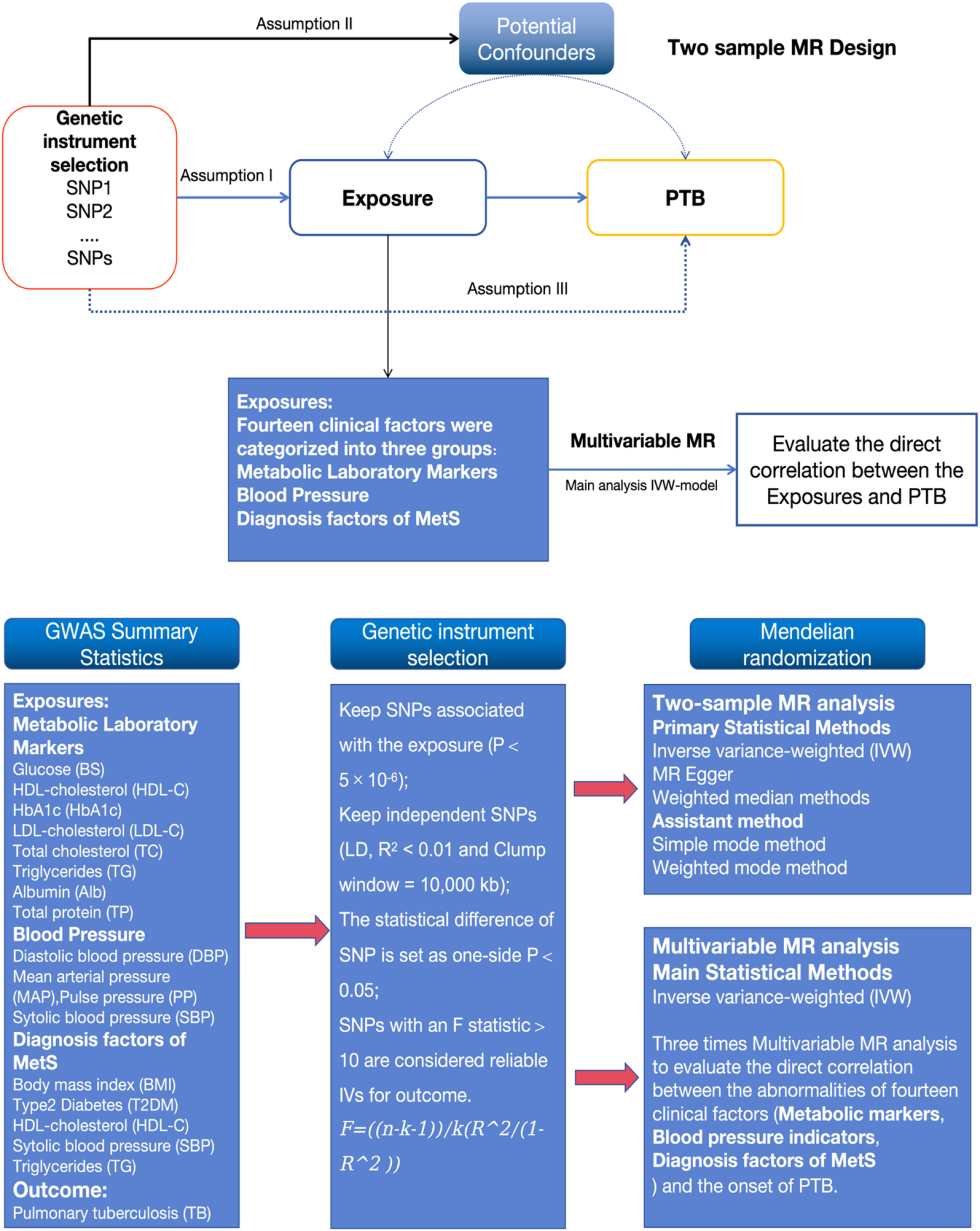
The flowchart of GWAS data sources and MR design (GWAS, genome-wide association studies; MR, Mendelian randomization; IVs, instrumental variables; SNPs, single-nucleotide polymorphisms. The GWAS summary statistics of this study were derived from the BioBank Japan Project (BBJ). Two-sample MR (TWMR) analyses are used to assess the causal association between metabolic disorder (MD) and pulmonary tuberculosis (PTB). Additionally, the direct effects on the risk of PTB were investigated through Multivariable MR. The 14 clinical factors were categorized into three groups, including metabolic laboratory markers, blood pressure, and the diagnosis factors of MetS. We used the threshold (*p* < 5 × 10^−6^) to choose the optimal IVs. To control linkage disequilibrium (LD) among the included IVs, the clumping process (*R*^2^ < 0.01 and clumping distance = 10,000 kb) was conducted to screen the included SNPs. Assumptions of TWMR 1: the instrumental variables of MetD should be closely related to PTB; assumption 2: the instrumental variables should not be associated with potential confounders, and assumption 3: the instrumental variables should affect the risk of PTB only through MetD and not through other alternative pathways. The primary method employed was the inverse variance-weighted (IVW) model in MVMR analysis.)

Figure 1 shows the study design of our MR analysis. The genome-wide association study (GWAS) summary statistics of this study were derived from the BioBank Japan Project (BBJ) Gene data [18]. BBJ is a pioneering genomic biobank that collaboratively collects DNA and serum samples from 12 medical institutions in Japan. To data, approximately 260,000 participants, primarily of Japanese ancestry, have been enrolled. Fourteen clinical factors related to MetD were selected as exposure variables in our study. The 14 clinical factors were categorized into three groups, including metabolic laboratory markers, blood pressure, and tMetS diagnostic factors. The summary statistics of pulmonary tuberculosis (TB) obtained from a GWAS meta-analysis of 7800 cases and 170,871 controls were used as outcome variables. The GWAS statistics of the 14 clinical factors and TB are summarized in Table 1. The Declaration of Helsinki statement was described in the original publications of these cohorts.

**Table 1:** Summary of the GWAS summary statistics

Phenotype	No. samples	No. cases	No. controls	PMID
Glucose (BS)	93,146.00	–	–	29403010
HDL-cholesterol (HDL-C)	70,657	–	–	29403010
HbA1c (HbA1c)	42,790	–	–	29403010
LDL-cholesterol (LDL-C)	72,866	–	–	29403010
Total cholesterol (TC)	128,305	–	–	29403010
Triglycerides (TG)	105,597	–	–	29403010
Albumin (Alb)	102,223	–	–	29403010
Total protein (TP)	113,509	–	–	29403010
Diastolic blood pressure (DBP)	136,615	–	–	29403010
Mean arterial pressure (MAP)	136,482	–	–	29403010
Pulse pressure (PP)	136,249	–	–	29403010
Systolic blood pressure (SBP)	136,597	–	–	29403010
Body mass index (BMI)	158,284	–	–	28892062
Type 2 diabetes	210,865	40,250	170,615	34594039
Pulmonary tuberculosis	178,671	7,800	170,871	34594039

PMID: ID of publication in PubMed

First, we conducted a two-sample Mendelian randomization analysis to evaluate the causal relationship between each of the 14 MetD factors and the outcome variable (TB). Second, we used the multivariable Mendelian randomization analysis method to study the variables that were included as exposure variables to evaluate the causal association between multiple genetic variations and the disease outcome when they coexisted.

### Genetic instrument selection

Single-nucleotide polymorphisms (SNPs) associated with 14 metabolic factors were selected as instrumental variables (IVs). In both the univariable and multivariable MR analyses, we used the threshold of *p* < 5 × 10^−6^ to choose the optimal IVs. We estimated linkage disequilibrium (LD, *R*^2^ < 0.01 and Clump window = 10,000 kb) between these SNPs for each metabolic factor. The F-statistics were calculated to assess the strength between metabolic factors and TB. The SNPs with a *P*-value < 0.05 and weak SNPs in the gene–outcome data were excluded from the IVs. Finally, the SNPs selected from 14 metabolic factors were confirmed to be unrelated to confounding factors after being queried in the phenoscanner database (http://www.phenoscanner.medschl.cam.ac.uk/). Detailed information on the IVs for metabolic factors and PTB is given in Additional file 1: Table S1.

**Fig. 2:**
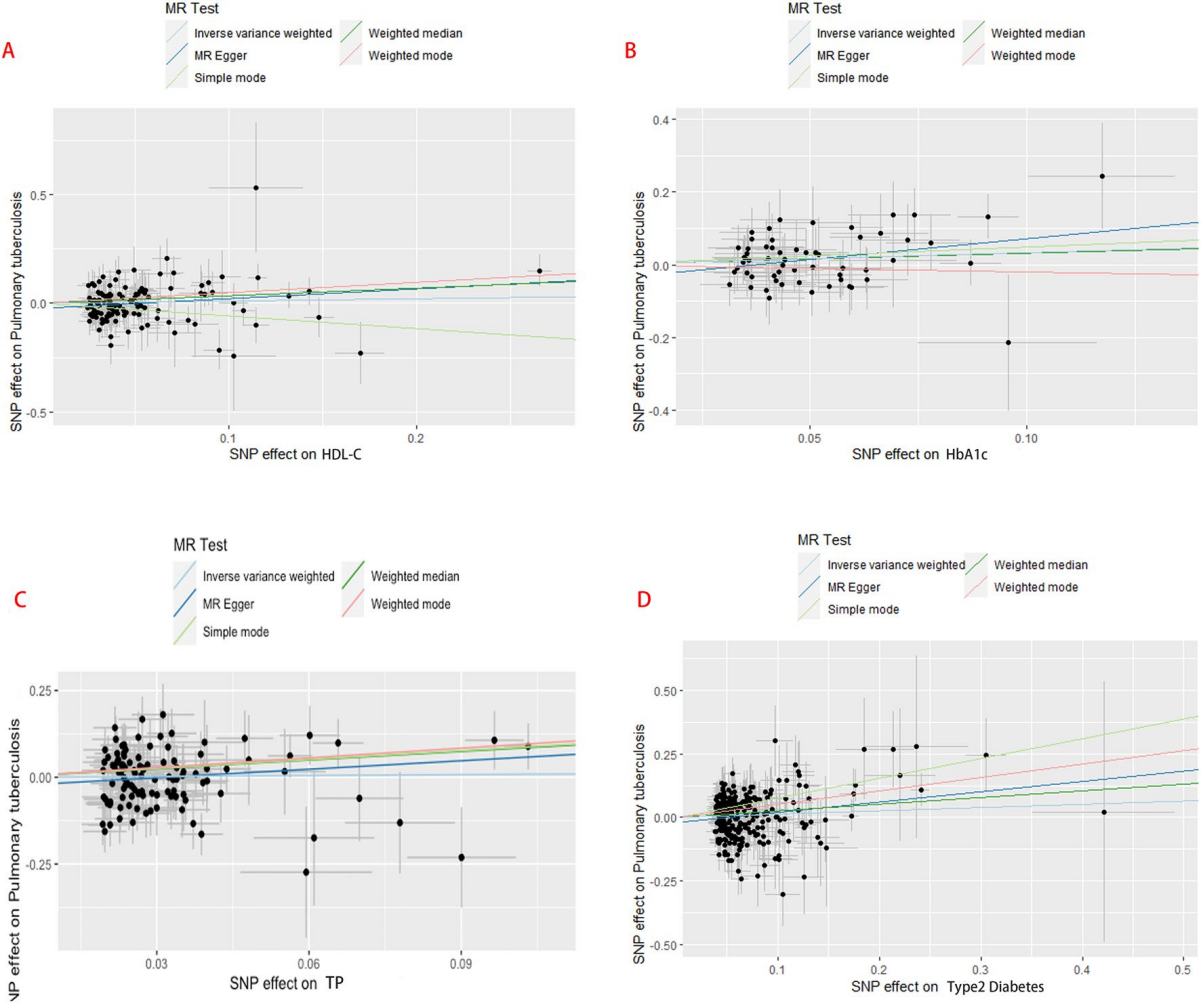
Scatter plots for the relationship between metabolic disorder and PTB. (Five methods (inverse variance–weighted method (light blue), MR Egger (blue); simple median method (light green), weighted median method (green), and weighted method (orange)) are used to assess the causal association between metabolic disorder (MD) and pulmonary tuberculosis (PTB) in the two-sample MR analysis.)

### Statistical analysis

#### Two-sample MR analysis

We first analyzed the causal relationships between the metabolic factors and TB by two-sample MR analysis. The inverse variance-weighted (IVW), MR Egger and weighted median methods were applied as the primary statistical methods to identify the consequence between metabolic factors and TB. We additionally applied the other method (the simple mode method, and weighted mode method) to examine the consistency of the MR results. The leave-one-out sensitivity test was used to evaluate the effect of a single IV on MR results. If the MR results estimated by the remaining IVs after removing a specific IV were significantly different from the total results, the MR results were sensitive to the IV. The *F*-statistics formula was $F=\frac{\left(n-k-1\right)}{k}(\frac{{R}^{2}}{1-{R}^{2}})$, where N is the sample size of each metabolic factors, K was the number of IVs associated with metabolic factors, and *R*^2^ was the proportion of the variability of the metabolic factors explained by IVs.

#### Multivariable MR analysis

The 14 clinical factors were categorized into three groups for further analysis. Glucose (BS), HDL-cholesterol (HDL-C), HbA1c (HbA1c), LDL-cholesterol (LDL-C), total cholesterol (TC), triglycerides (TG), albumin (Alb), and total protein (TP) were classified as metabolic laboratory markers. Blood pressure was defined as diastolic blood pressure (DBP), mean arterial pressure (MAP), pulse pressure (PP), and systolic blood pressure (SBP). According to the guidelines of the JCCDS, body mass index (BMI), T2DM, high-density lipoprotein (HDL)-C, systolic blood pressure (SBP), and triglyceride (TG) levels were selected as the diagnostic factors for MetS. Multivariable MR analysis was performed three times to evaluate the direct correlation between the abnormalities of 14 clinical factors and the onset of PTB. We used the standard of *p* < 5 × 10^6 to screen the optimal independent variables (IVs). For each factor, we estimated the linkage disequilibrium (LD) between these SNPs (*R*^2^ < 0.01, Clump window = 10,000 kb), and the IVW model was used as the main method.

We additionally evaluated the sensitivity of this MR analysis. The heterogeneity test was performed to assess the difference between each IV (Cochran's Q) by MR-Egger regression. *P* < 0.05 according to Cochran's Q test indicated heterogeneity. The MR-PRESSO global test was applied to evaluate horizontal pleiotropy between multiple IVs. Finally, the statistical power of the two-sample MR analysis was estimated by an online tool (https://shiny.cnsgenomics.com/mRnd/). R version 4.3.0 software, the “TwoSampleMR” package, the “MendelianRandomization” package and the “MVMR” package were used to perform this MR analysis.

## Results

### Two-sample Mendelian randomization (TSMR)

In the two-sample MR analysis, 14 clinical factors, including metabolic laboratory markers, blood pressure, and the diagnostic factors of MetS, were categorized into three groups. The number of SNPs selected as instrumental variables (IVs) ranged from 43 to 273, and the F-statistics for each SNP are summarized in Additional file 1: Table S1. We investigated the causal association between metabolic disorders (MDs) and pulmonary tuberculous (PTB) using five models. The inverse variance-weighted (IVW) method, MR-Egger test, and weighted median test were used as the primary statistical methods to identify differences between MD and PTB patients, and the results are shown in Table 2. After that, four factors (HDL-C, HbA1c, TP, and DM) had at least one method to obtain a *P* value < 0.05. The exposure variables HDL-C (MR Egger, *P* = 0.032, OR 1.565; weighted median, *P* = 0.056, OR 1.424), HbA1c (inverse variance weighted, *P* = 0.043, OR 1.400), TP (weighted median, *P* = 0.018, OR 2.292), and DM (MR Egger, *P* = 0.011, OR 1.502; weighted median, *P* = 0.018, OR 1.298; inverse variance weighted, *P* = 0.055, OR 1.136) were causally associated with active PTB (Fig 2). No horizontal pleiotropy was identified in the IVs selected from four metabolic factors by MR‒Egger regression (P for Cochran’s Q). The MR-PRESSO test showed no significant heterogeneity in the enrolled SNPs. The results of the heterogeneity and horizontal pleiotropy tests are shown in Table 3.

**Table 2:** The results of two-sample MR analysis

Phenotype	Method	nSNP	Beta	SE	*P*-value	OR	95% CI
**BS**	MREgger	43	0.094	1.111	0.933	1.099	0.125–9.689
	Weighted median	43	0.012	0.407	0.976	1.012	0.456–2.248
	Inverse variance weighted	43	0.292	0.290	0.315	1.339	0.758–2.366
HDL-C	MR Egger	124	0.448	0.207	0.032	1.565	1.044–2.347
	Weighted median	124	0.353	0.185	0.056	1.424	0.99–2.047
	Inverse variance weighted	124	0.106	0.113	0.346	1.112	0.892–1.387
HbA1c	MR Egger	59	1.145	0.607	0.064	3.143	0.956–10.332
	Weighted median	59	0.309	0.261	0.236	1.362	0.817–2.271
	Inverse variance weighted	59	0.337	0.181	0.043	1.400	0.982–1.997
LDL-C	MR Egger	76	− 0.179	0.355	0.615	0.836	0.417–1.675
	Weighted median	76	− 0.092	0.271	0.734	0.912	0.536–1.552
	Inverse variance weighted	76	− 0.291	0.188	0.123	0.748	0.517–1.082
TC	MR Egger	120	0.135	0.489	0.782	1.145	0.439–2.983
	Weighted median	120	− 0.157	0.311	0.613	0.854	0.465–1.571
	Inverse variance weighted	120	− 0.226	0.211	0.284	0.798	0.527–1.206
TG	MR Egger	101	0.094	0.233	0.687	1.098	0.696–1.734
	Weighted median	101	− 0.143	0.223	0.522	0.867	0.56–1.342
	Inverse variance weighted	101	0.158	0.144	0.273	1.171	0.883–1.553
Albumin (Alb)	MR Egger	50	0.693	0.920	0.455	2.000	0.329–12.147
	Weighted median	50	− 0.261	0.489	0.594	0.771	0.295–2.01
	Inverse variance weighted	50	− 0.328	0.310	0.290	0.721	0.393–1.322
TP	MR Egger	114	0.818	0.534	0.128	2.265	0.796–6.449
	Weighted median	114	0.829	0.349	0.018	2.292	1.156–4.543
	Inverse variance weighted	114	0.086	0.218	0.693	1.090	0.711–1.67
DBP	MR Egger	78	0.824	1.333	0.539	2.279	0.167–31.074
	Weighted median	78	0.515	0.488	0.291	1.674	0.643–4.356
	Inverse variance weighted	78	0.416	0.332	0.210	1.516	0.791–2.903
MAP	MR Egger	100	0.692	1.002	0.491	1.997	0.28–14.232
	Weighted median	100	0.090	0.409	0.826	1.094	0.491–2.441
	Inverse variance weighted	100	0.182	0.268	0.497	1.200	0.709–2.031
PP	MR Egger	48	0.813	1.923	0.674	2.255	0.052–97.741
	Weighted median	48	0.163	0.586	0.780	1.178	0.374–3.711
	Inverse variance weighted	48	0.656	0.412	0.112	1.927	0.859–4.326
SBP	MR Egger	94	0.734	1.058	0.490	2.083	0.262–16.576
	Weighted median	94	0.015	0.406	0.970	1.015	0.458–2.252
	Inverse variance weighted	94	0.176	0.278	0.527	1.192	0.691–2.057
BMI	MR Egger	246	− 0.751	0.603	0.214	0.472	0.145–1.539
	Weighted median	246	− 0.333	0.302	0.270	0.717	0.396–1.296
	Inverse variance weighted	246	− 0.289	0.186	0.120	0.749	0.52–1.079
DM	MR Egger	273	0.407	0.159	0.011	1.502	1.1–2.05
	Weighted median	273	0.261	0.110	0.018	1.298	1.046–1.61
	Inverse variance weighted	273	0.128	0.067	0.055	1.136	0.997–1.295

BS: Glucose; HDL-C: HDL-cholesterol; HbA1c: HbA1c; LDL-C: LDL-cholesterol; TC: total cholesterol; TG: triglycerides; Alb: albumin; TP: total protein; DBP: diastolic blood pressure; MAP: mean arterial pressure; PP: pulse pressure; SBP: systolic blood pressure; BMI: body mass index; DM: Type 2 diabetes; nSNP: numbers of single-nucleotide polymorphisms; SE: standard Error; OR: odds ratio; 95% CI: confidence Interval

**Table 3:** The results of the heterogeneity and horizontal pleiotropy test

Phenotype	IVs	Cochran's Q	*P* pleiotropy	*P* heterogeneity
Glucose (BS)	43	26.851	0.934	0.957
HDL-cholesterol (HDL-C)	124	110.677	0.672	0.76
HbA1c (HbA1c)	59	39.568	0.918	0.962
LDL-cholesterol (LDL-C)	76	87.816	0.125	0.13
Total cholesterol (TC)	120	148.75	0.263	0.129
Triglycerides (TG)	101	85.548	0.84	0.83
Albumin (Alb)	50	42.313	0.495	0.704
Total protein (TP)	114	124.792	0.084	0.193
Diastolic blood pressure (DBP)	78	82.988	0.357	0.273
Mean arterial pressure (MAP)	100	93.597	0.636	0.607
Pulse pressure (PP)	48	40.291	0.709	0.709
Systolic blood pressure (SBP)	94	67.964	0.987	0.972
Body mass index (BMI)	246	251.263	0.399	0.361
Type 2 diabetes	273	283.159	0.179	0.293

*P* pleiotropy = *P*-values for pleiotropy were derived from MR-Egger test and *P*-value < 0.05 indicates a possible pleiotropic effect. *P* heterogeneity = *P*-values for distortion were derived from MR-PRESSO test and *P*-value < 0.05 indicates a possible Heterogeneity effect

### Multivariable Mendelian randomization (MVMR)

**Fig. 3:**
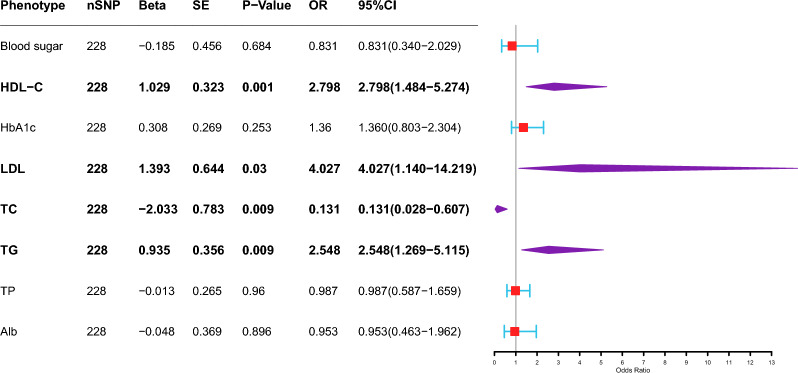
Association of genetically predicted metabolic laboratory markers with risk of PTB. (The inverse variance-weighted (IVW) method is used for the univariable MR analysis. The range of the abscissa of the forest plot is set as 1–13. BS: glucose; HDL-C: HDL-cholesterol; HbA1c: HbA1c; LDL-C: LDL-cholesterol; TC: total cholesterol; TG: triglycerides; Alb: albumin; TP: total protein)

**Fig. 4:**
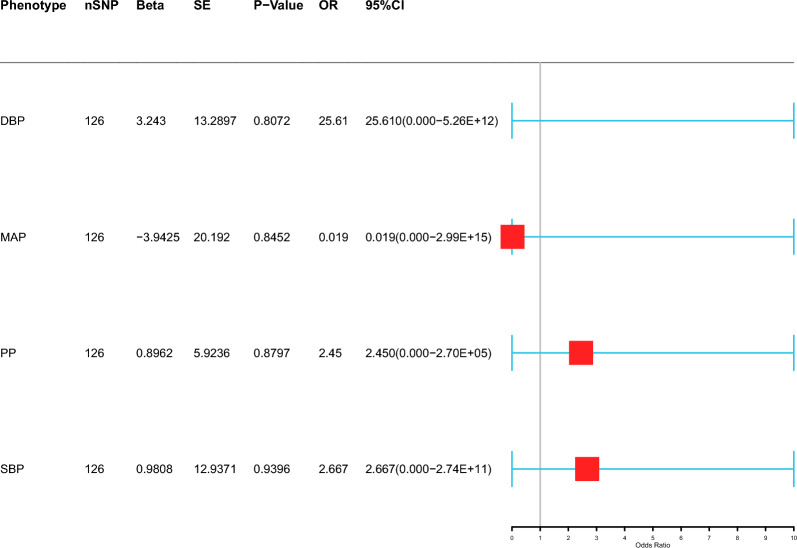
Association of genetically predicted blood pressure with risk of PTB. (The inverse variance-weighted (IVW) method is used for the univariable MR analysis. The range of the abscissa of the forest plot is set as 1–10. DBP: Diastolic blood pressure; MAP: mean arterial pressure; PP: pulse pressure; SBP: systolic blood pressure)

**Fig. 5:**
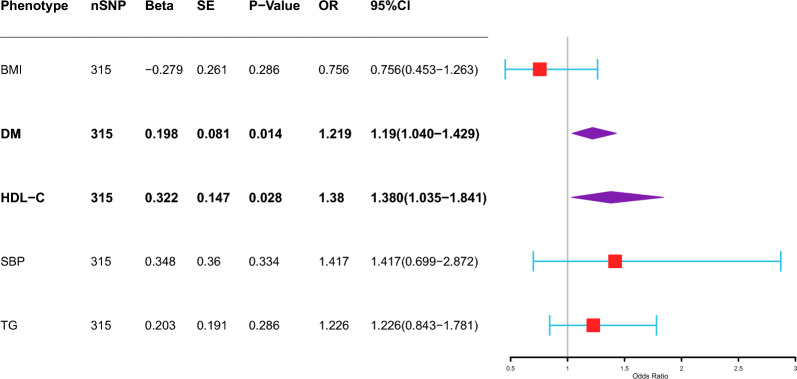
Association of genetically predicted metabolic syndrome with risk of PTB. (The inverse variance-weighted (IVW) method is used for the univariable MR analysis. The range of the abscissa of the forest plot is set as 1–3. BMI: Body mass index; DM: Type 2 diabetes; HDL-C: HDL-cholesterol; SBP: systolic blood pressure; TG: triglycerides)

Multivariable Mendelian randomization (MVMR) was used to evaluate the associations between eight metabolic laboratory markers and PTB (Fig. 3). After adjusting for other laboratory markers, three indicators were positively correlated with the occurrence of PTB. HDL-C (IVW: OR 2.798, 95% CI 1.484–5.274, *P* = 0.001), LDL (IVW: OR = 4.027, 95% CI 1.140–14.219, *P* = 0.03) and TG (IVW: OR 2.548, 95% CI 1.269–5.115, *P* = 0.009) were positively correlated with the occurrence of PTB. TC (OR 0.131, 95% CI 0.028–0.607, *P* = 0.009) was negatively correlated with the occurrence of PTB. No heterogeneity of the summary estimates was estimated using Cochran’s Q test (*P* value = 0.6619). There was no significant association between blood pressure and PTB according to the MVMR analysis (Fig. 4). BMI, DM, HDL-C, SBP, and TG were used as diagnostic factors for metabolic syndrome. DM (IVW, OR = 1.219, 95% CI 1.040–1.429 *P* = 0.014) and HDL-C (IVW, OR = 1.380, 95% CI 1.035–1.841, *P* = 0.028) were directly correlated with the occurrence of PTB (Fig. 5). No heterogeneity of the summary estimates was estimated using Cochran’s Q test (*P* value = 0.297).

## Discussion

Pulmonary tuberculosis (PTB) patients with endocrine or metabolic disorders (MetDs) are not uncommon in China, and the basis for these abnormalities is complex [13]. Diabetes, hypertension and dyslipidemia are the most common metabolic disorders. Significant correlations have been demonstrated between multiple metabolic diseases. Epidemiological studies have shown that cardiovascular disease (CVD) patients with DM have a greater incidence of hypertension [19]. A multicenter clinical trial indicated that the risk of complications, including hypertension and dyslipidemia, in young DM patients increased steadily over time [20]. The Chinese Diabetes Society (CDS) and JCDCG recommend that individuals with one of three or more factors, obesity, BMI ≥ 25.0 kg/m^2^, DM, hypertension, or dyslipidemia, be diagnosed with metabolic syndrome (MetS) [21]. Through a literature review, we found that the associations between metabolic disorders (MetDs) or metabolic syndrome (MetS) and pulmonary tuberculosis (PTB) are poorly understood.

Mendelian randomization (MR) is a scientific approach that establishes causal relationships between modifiable risk factors and diseases by utilizing genetic variants in natural experiments. This method provides valuable insights into potential causal connections, enabling researchers to make more informed decisions regarding preventive measures and therapeutic interventions. Although MR only focuses on the genetic components of each factor and ignores the environmental components, compared with traditional observational studies, MR is less affected by confounding factors and reverse causality [22]. In this study, the causal relationships between the metabolic factors and PTB were confirmed by two-sample MR analysis. The 14 clinical factors were categorized into three groups, including metabolic laboratory markers, blood pressure, and MetS diagnostic factors. Four factors, HDL-C, HbA1c, TP, and DM, are causally correlated with the incidence of active TB. Multivariable Mendelian randomization (MVMR) is an extension of Mendelian randomization that uses genetic variants associated with multiple potentially related exposures to estimate the direct effects of each exposure on a single outcome [23]. Multivariable MR analysis was performed three times to evaluate the direct correlation between the abnormalities of 14 clinical factors (metabolic markers, blood pressure indicators, and diagnostic factors for MetS) and the onset of PTB. After correcting for other metabolic factors, HDL-C, LDL and TG were positively correlated with the occurrence of PTB. TC was negatively correlated with the occurrence of PTB. Consistent with the results of the two-sample MR analysis, there was no significant association between blood pressure factors and PTB. We subsequently selected BMI, DM, HDL-C, SBP, and TG as the diagnostic factors for metabolic syndrome. DM and HDL-C were 1.219 (95% CI 1.040–1.429, *P* = 0.014) and 1.380 (95% CI 1.035–1.841, *P* = 0.028), respectively, for the occurrence of PTB.

Hemoglobin A1c (HbA1c) is one of the criteria which is used for the diagnosis and management of DM [24]. Most patients infected with *Mycobacterium tuberculosis* are asymptomatic, which is defined as latent tuberculosis infection (LTBI). The screening of LTBI is a critical component of the End TB Strategy proposed by the WHO [25]. Identifying risk factors for the reactivation of LTBI through rational statistical hypotheses holds significant implications for tuberculosis prevention and control efforts. T2DM is indicated to have a significant association with the incidence of LTBI. A study based on US National data reported that T2DM is positively correlated with LTBI (OR 1.90, 95% CI 1.15–3.14) [26]. More critically, T2DM increases the progression from LTBI to active TB, and the mechanism of susceptibility to TB among T2DM patients is poorly understood [27]. These studies were all retrospective cohort studies, and the reliability of their results was lower than that of randomized controlled studies. A well-conducted MR study is less likely to be affected by confounding factors than a conventional observational study. DM and pulmonary tuberculosis showed a direct causal relationship in our MR study. Restrepo et al. [28] reported that the association of TB with monocytes was significantly lower in DM patients (19.2 ± 6.1) than in non-DM patients (27.5 ± 7.9; *P* = 0.02). Blood monocytes migrate to the lung upon MTB infection and then differentiate into macrophages and dendritic cells (DCs). MTB resists killing by alveolar macrophages and leads to the death of these cells [29]. Insulin resistance promotes M2 macrophage polarization via NF-kappa B, AP1, and other signal-dependent transcription factors and affects the host innate immune response to tuberculosis [30]. DCs play a pivotal role in linking innate and adaptive immune responses. Kumar et al.'s study indicated that the frequencies of both myeloid DCs and plasmacytoid DCs in TB patients with DM are significantly lower than those in patients without DM. In addition, the cellular subset distributions of T cells, B cells, DCs, and macrophages are altered in active TB patients with DM [31]. DM is associated with increased number of antigen-stimulated CD8+ T cells expressing IFN-γ, IL-2, and IL-17F in PTB patients [32]. In summary, the relationship between glucose metabolism disorders and TB risk has been confirmed in clinical and basic research.

Abnormal lipid metabolism (HDL-C, HDL-C, LDL, TG, and TC) was also confirmed to have a direct causal relationship with the occurrence of PTB in our study. Consistent with our results, low total cholesterol (TC) levels are guaranteed to be associated with a high risk of PTB in South Korea [15]. A case–control study reported that the baseline TG levels detected by unbiased lipidomics in TB patients with treatment failure were significantly lower than those in control individuals. Baseline TG levels are positively associated with treatment failure among active PTB patients [33]. “Omics” is a new approach to the study of TB susceptibility, pathogenesis and treatment outcomes. A case–control study using unbiased lipid omics confirmed that oxylipins, cholesteryl esters, ceramides, sphingomyelins, diacylglycerol and triacylglycerol are associated with TB treatment failure [33]. UPLC-QToF MS revealed that cholesterol ester (CE), monoacylglycerols, and phosphatidylcholine (PC) were associated with host immune responses in active TB patients [34]. Han et al. [35] speculated that M. tuberculosis infection may regulate lipid metabolism in TB patients and may promote host-assisted bacterial degradation of phospholipids and the accumulation of cholesterol esters. However, the impact of different types of dyslipidemia on TB susceptibility is still poorly understood. In the absence of multicenter randomized controlled studies to analyze the potential association between metabolic driving factors and tuberculosis infection, our MR study provides powerful evidence-based medicine for the prevention and management of TB.

In conclusion, this MR study demonstrated that metabolic disorders, mainly hyperglycemia and dyslipidemia, are associated with the incidence of active pulmonary tuberculosis. Unlike the confirmed direct causal relationship between type 2 diabetes and pulmonary tuberculosis, the impact of dyslipidemia on the progression from tuberculosis infection to active tuberculosis requires further investigation.

## Limitations

This study had several limitations. First, East Asia is a region at high risk for TB, and our study analyzed only the East Asian population. Although the incidence of tuberculosis is lower in Europe, the incidence of metabolic disorders is more severe. We need to obtain GWAS data from different countries and regions in the future to validate the results of our study. Second, MR itself has certain limitations; it profiles a genetic component of each trait rather than its environmental component. The causal relationship between exposure and outcome factors, confirmed by MR analysis, is difficult to confirm epidemiologically. For example, in our study, it was confirmed that abnormal metabolism of some lipids increases the risk of TB development. It is difficult to confirm epidemiologically whether these abnormalities in lipid metabolism are a cause or a consequence of TB development.

### Supplementary Information

**Additional file 1.** Detailed information on metabolic factors as IVs.

## Data Availability

Detailed information on those IVs for metabolic factors and PTB is given in Additional file 1: Table S1.
